# Preoperative prediction of non-invasive follicular thyroid neoplasm with papillary-like nuclear features: a Canadian single-Centre experience

**DOI:** 10.1186/s40463-019-0397-9

**Published:** 2020-01-02

**Authors:** Vincent Larouche, Marc Philippe Pusztaszeri, Sabin Filimon, Richard Payne, Michael Hier, Michael Tamilia

**Affiliations:** 1 Division of Endocrinology and Metabolism, Jewish General Hospital, 3755, Chemin de la Côte-Sainte-Catherine, Montréal, QC H3Y 1E2 Canada; 2 Division of Pathology, Jewish General Hospital, 3755, Chemin de la Côte-Sainte-Catherine, Montréal, QC H3Y 1E2 Canada; 3 Internal Medicine Residency Training Program, McGill University, 3755, Chemin de la Côte-Sainte-Catherine, Montréal, QC H3Y 1E2 Canada; 4 Division of Oto-Rhino-Laryngology, Jewish General Hospital, 3755, Chemin de la Côte-Sainte-Catherine, Montréal, QC H3Y 1E2 Canada; 5 Division of Endocrinology and Metabolism, Jewish General Hospital, 3755, Chemin de la Côte-Sainte-Catherine, Montréal, QC H3Y 1E2 Canada

**Keywords:** NIFTP, Preoperative, Prediction, Ultrasound, Laboratory, Clinical, Features

## Abstract

**Background:**

An international group of experts recommended reclassifying non-invasive follicular variant of papillary thyroid cancers (FVPTC) as ‘non-invasive follicular thyroid neoplasm with papillary-like nuclear features’ (NIFTP) in April 2016. The purpose of this study was to establish preoperative clinical, laboratory, ultrasonographic, and cytological variables, which can differentiate NIFTP from FVPTC.

**Methods:**

We conducted a retrospective chart review of consecutive patients from a single institution evaluated between January 2012 and December 2017. 203 adult patients underwent lobectomy or total thyroidectomy for a FVPTC during that period. Each patient’s medical chart was reviewed and information on pre-operative variables was recorded. An expert pathologist reviewed all surgical specimens and reclassified a subset of FVPTC as NIFTP according to the specific criteria.

**Results:**

Overall, 44 patients were included in the NIFTP group and 159 in the non-NIFTP group. Mean age was 50.1 years in the NIFTP group and 50.7 in the non-NIFTP group. Most patients were female (86.4% (38/44) in the NIFTP group vs 79.8% (127/159) in the non-NIFTP group). More patients underwent lobectomy in the NIFTP group (50% (22/44) vs 16.4% (26/159) in the non-NIFTP group, p = < 0.0001). Less patients received radioactive iodine in the NIFTP group (31.8% (14/44) vs 52.2% (83/159) in the non-NIFTP group, *p* = 0.0177). Preoperative thyroglobulin levels were lower in NIFTP patients (Median 25.55 mcg/L +/− 67.8 vs 76.06 mcg/L +/− 119.8 in Non-NIFTP, *p* = 0.0104). NIFTP nodules were smaller (Mean size 22.97 mm +/− 12.3 vs 25.88 mm +/− 11.2 for non-NIFTP, *p* = 0.0448) and more often solid than non-NIFTP (93.2% (41/44) vs 74.8% (119/159) for non-NIFTP, *p* = 0.0067). 2017 ACR TIRADS nodule category of 1–4 on ultrasound had a negative predictive value and a sensitivity of 100% for NIFTP. ROC Curve Analysis demonstrated that a preoperative thyroglobulin level of 31.3 mcg/L had a sensitivity of 75% and a specificity of 62.5% to differentiate NIFTP from non-NIFTP cancers.

**Conclusion:**

Lower preoperative thyroglobulin levels, smaller nodule size, solid texture and 2017 ACR TIRADS Category of 1–4 are more strongly associated with NIFTP than FVPTC and can favour less invasive surgical options such as lobectomy.

## Introduction

Historically, classical-variant papillary thyroid cancer (CVPTC) has been the most prevalent histological subtype of papillary thyroid cancer, however, several reports have cited a rise in frequency of follicular-variant of PTC (FVPTC) ranging from 9 to 22.5% [1, 2]. A recent multi-institutional study examined 109 patients with non-invasive encapsulated FVPTC and found that none of these patients developed a recurrence, with a follow-up time varying between 10 and 26 years [3]. Based on this indolent clinical course, in April 2016, an international multidisciplinary group of experts recommended reclassifying these tumours as ‘non-invasive follicular thyroid neoplasm with papillary-like nuclear features’ (NIFTP). The purpose of this reclassification was to reduce the clinical and psychological consequences associated with the diagnosis of cancer. Specifically, NIFTP are diagnosed with 6 strict pathological criteria; encapsulation or clear demarcation, follicular growth pattern with < 1% papillae, absence of psammomma bodies and < 30% solid/trabecular/insular growth pattern, nuclear score 2–3, absence of vascular or capsular invasion, absence of tumour necrosis and absence of a high mitotic activity [3, 4]. As per current American Thyroid Association practice guidelines, NIFTP lesions nonetheless warrant excision by lobectomy to exclude an invasive FVPTC, CVPTC, or other thyroid malignancy [5, 6]. Once the diagnosis of NIFTP is made histologically, however, further therapy such as completion thyroidectomy and/or radioactive iodine therapy is not warranted and a more conservative approach is recommended. Indeed, as individualized care of thyroid cancer patients is promoted, avoiding overtreatment of patients with low risk disease is recommended [6]. The purpose of this study was to establish preoperative clinical, laboratory, ultrasonographic and cytological variables in NIFTP patients, which can help differentiate these patients from those with non-NIFTP follicular cancers (i.e., FVPTC).

## Material and methods

We conducted a retrospective chart review of patients evaluated at the Thyroid Cancer Clinic of the Endocrinology Division of a university-affiliated institution from January 2012 to December 2017. The institutional ethics review board approved this retrospective study and informed consent was waived. From a prospectively maintained tumour registry, we identified consecutive adult patients who underwent lobectomy or total thyroidectomy for a NIFTP or a non-NIFTP follicular lesion (invasive FVPTC or non-invasive FVPTC not meeting NIFTP criteria [3]). Each patient’s medical record was reviewed and information on pre-operative variables including clinical characteristics such as age at diagnosis and gender, laboratory values (thyrotropin (TSH), thyroglobulin (Tg), and anti-thyroglobulin antibodies, (anti-Tg-Ab)), ultrasonographic features, fine needle aspiration (FNA) cytology (Bethesda category, BC), Hector Batifora mesothelial 1 (HBME-1) immunostaining and final operative pathology were recorded. Exclusion criteria included patients younger than 18 years, those with a thyroid nodule FNA cytology showing Bethesda categories I-II, those with a final pathology showing CVPTC or its variants, and other types of thyroid cancer (anaplastic, lymphoma, medullary, metastases). The exclusion of nodules with FNA cytology showing Bethesda I and II was a decision based on the fact that the vast majority of NIFTP nodules (more than 90%) are in the intermediate categories, therefore authors wanted to focus on such categories (Bethesda III and higher) in the analysis. Considering that NIFTP lesions started being recorded on pathology reports as of April 2016 at our institution, a pathologist with expertise in thyroid histology, MPP, reviewed all surgical specimens from prior years, re-analysed and re-classified them into either NIFTP, or FVPTC not meeting the diagnostic criteria of NIFTP. This represented 144 cases of FVPTC, of which 33 were reclassified as NIFTP and the other cases remained in the non-NIFTP group.

### Biochemistry

Thyrotropin was measured by an electrochemiluminescence immunometric assay (TSH Thyrotropin Cobas assay (RocheDiagnostics). The assay has a functional sensitivity of 0.01 mIU/L and a reference value established in-house ranging from 0.4 to 4.5 mIU/L. Thyroglogulin (Tg) was measured by an immunometric assay (Immulite 2000 Thyroglobulin; Siemens, Los Angeles, CA) with a functional sensitivity of 0.9 lg/L. Anti-Tg Ab were determined by a chemiluminescent assay (Immulite 2000 Anti-Tg Ab; Siemens) and considered negative when < 20 IU/mL.

### Neck sonography

Sonography was performed by the same operator (MT) with a linear multi-frequency 12–14 MHz transducer (General Electric, Fairfield, CT) for morphological analysis (B-mode). Initially, the thyroid nodule was characterized and the central and lateral compartments were examined for suspicious lesions. The nodule of interest was biopsied with 22 or 23-gauge needle mounted on a 10 ml syringe under ultrasound guidance with the material processed for cytology and cellblocks for immunocytochemistry. For the purpose of this study, the stored images were retrieved and the following sonographic features of the nodules were reviewed (i.e composition- cystic, complex, or solid, echogenicity- iso-, hyper- or hypo-echoic, orientation – taller-than-wide, margins- ill-defined, irregular (lobulated, spiculated), or regular, and presence of echogenic foci- none, macro- or micro-calcifications). The weighted scores of these features were combined and the total value classified into one of six categories according to the American College of Radiology (ACR) Thyroid Imaging Reporting and Data System (TI-RADS) guidelines (i.e. TI-RADS 1-benign, TI-RADS 2 –no suspicion, TI-RADS 3 -low suspicion, TI-RADS 4 -moderate suspicion, and TI-RADS 5 -high suspicion [7].

### Statistical analysis

SPSS Version 12.0 (SPSS, Chicago, III) was used for statistical analysis. All variables in two groups (NIFTP vs non-NIFTP) were compared. For all characteristics, continuous variables (age, maximal diameter, preoperative thyroglobulin level and secretory index) were analysed using a two-tailed Mann-Whitney test and categorical variables were analysed using a two-tailed Fisher’s exact test or Chi Square Test. In general, a *p* value < 0.05 was considered statistically significant. Diagnostic test evaluation calculations were performed to determine the sensitivity, specificity, negative and positive predictive values of Bethesda cytology categories on FNA, ACR TIRADS Ultrasound categories or preoperative thyroglobulin levels whole distribution quartiles or combinations to distinguish NIFTP from non-NIFTP follicular cancers. Receiver operator characteristic (ROC) curve analysis was used for preoperative thyroglobulin level. The optimal cut-off values were defined as the value at which the sum of the sensitivity and specificity was maximized.

## Results

Of the entire cohort of patients (*n* = 318) who underwent thyroid surgery, 222 (69.8%) patients with a follicular-patterned thyroid tumour were retained for the study. Nineteen cases of follicular adenoma were also excluded from the analysis, as it is a benign tumour and our focus was on comparing and contrasting NIFTP from the more aggressive forms of FVPTC. Author MPP reviewed all cases of follicular adenomas and all pathological diagnoses remained the same, based on negative immunohistochemistry stains for HBME-1, Galectin-3 and CK19. Of the 203 patients, 44 (21.7%) patients were included in the NIFTP group and 159 (78.3%) patients were included in the non-NIFTP group (148 minimally invasive encapsulated FVPTC and 11 infiltrative FVPTC). The comparison of the demographic characteristics and clinical management between the NIFTP and the non-NIFTP groups are summarized in Table 1. The mean age was 50.1 years (standard deviation SD 13.6, Range 28–84) in the NIFTP group and 50.7 (SD 14.9, Range 18–82) in the non-NIFTP group. The majority of patients were female in both groups (86.4 and 79.8% in the NIFTP group and the non-NIFTP group respectively). In terms of surgical management, lobectomy was performed more often in NIFTP patients than in the non-NIFTP patients (50 and 16.4% respectively, *p* <  0.0001). Fewer patients received radioactive iodine therapy in the NIFTP group (31.8% vs 52.2%, *p* = 0.0177). There was no significant difference between groups with regards to American Joint Committee on Cancer (AJCC) Tumour Node Metastasis (TNM) Staging (7th edition) [8] or ATA Initial Risk Stratification system categories [5].

**Table 1:** Demographic characteristics and management of NIFTP vs non-NIFTP groups

Characteristics	NIFTP	Non-NIFTP	Total	*P* value
	*n* = 44	*n* = 159	*n* = 203	
Age (years), mean (SD)	50.1 +/− 13.6	50.7 +/− 14.9	50.6 +/− 14.6	0.8099^a^
Female gender, n (%)	38 (86.4)	127 (79.8)	165 (81.3)	0.3889^b^
Surgery Type				< 0.0001^c *^
Lobectomy, n (%)	22 (50)	26 (16.4)	48 (23.6)	
All other surgeries, n (%)	22 (50)	133 (83.6)	155 (76.4)	
RAI Therapy, n (%)	14 (31.8)	83 (52.2)	97 (47.8)	0.0177^b *^
AJCC TNM Staging, n (%)				0.5349^c^
pT1	22 (50)	70 (44)	92 (45.3)	0.5349
pT2	17 (38.6)	60 (37.8)	77 (38.0)	0.5349
pT3	5 (11.4)	29 (18.2)	34 (16.7)	0.5349
N0	44 (100)	149 (93.7)	193 (95.1)	0.1225
N1a	0	10 (6.3)	10 (4.9)	0.1225
ATA risk, n (%)				0.1712^b^
I	44 (100)	147 (92.6)	191 (94)	
II	0	6 (3.7)	6 (3)	
III	0	6 (3.7)	6 (3)	

^a^two-tailed Mann-Whitney test

^b^two-tailed Fisher’s exact test

^c^chi-square test

^*^statistically significant (*P* < 0.05)

The comparison of the laboratory and cytological characteristics between the NIFTP and non-NIFTP groups are summarized in Table 2. Preoperative serum thyroglobulin levels, in the absence of anti-Tg antibodies, was significantly lower in NIFTP than in non-NIFTP patients (median 25.55 mcg/L +/− 67.8 (IQR) vs 76.06 mcg/L +/− 111.2, *p* = 0.0104) The thyroglobulin index (mcg/L/cm), defined as the preoperative thyroglobulin level (mcg/L) divided by the largest diameter (cm) on ultrasound was also significantly lower in NIFTP patients (median 11.34 mcg/L/cm +/− 20.2 vs 32.0 +/− 53.9, *p* = 0.0089). Although not statistically significant, the non-NIFTP group of patients trended for higher BC categories and only 1 (2.3%) NIFTP patient was in the BC-VI (Malignant) category. There was no significant difference between both groups in the preoperative TSH, Bethesda category on FNA or HBME-1 immunocytochemistry.

**Table 2:** Laboratory and cytological characteristics of NIFTP vs non-NIFTP groups

Characteristics	NIFTP	Non-NIFTP	Total	*P* value
Normal Preoperative TSH (0.4–4.50 IU/L), n (%)	43 (100)	152 (96.2)	195 (97)	0.3447^b^
Pre-operative Thyroglobulin Level (mcg/L), Median +/− IQR	*n* = 24 25.6 +/− 67.8	*n* = 92 76.1 +/− 119.8	67.4 +/− 111.2	0.0104^a *^
Thyroglobulin Index (mcg/L/cm), Median +/− IQR	n = 24 11.3 +/− 20.2	n = 92 32.0 +/− 53.9	28.1 +/− 47.7	0.0089^a *^
Bethesda Category				
III	28 (63.6)	80 (50.3)	108 (53.2)	0.0653^c^
IV	1 (2.3)	9 (5.7)	10 (4.9)	
V	14 (31.8)	46 (28.9)	60 (29.6)	
VI	1 (2.3)	24 (15.1)	25 (12.3)	
HBME-1 Positive	23 (52.3)	69 (43.4)	92 (45.3)	0.3097^b^

^a^two-tailed Mann-Whitney test

^b^two-tailed Fisher’s exact test

^c^chi-square test

^*^statistically significant (*P* < 0.05)

More specifically, 14/44 (31.8%) NIFTP cases had a thyroid nodule FNA cytology consistent with Bethesda V (Suspicious for malignancy). Of these, 8 underwent lobectomy and 6 underwent total thyroidectomy. 9 were diagnosed before and 5 after 2016.

The comparison of the ultrasound features between the NIFTP and non-NIFTP groups are summarized in Table 3. NIFTP nodules were significantly smaller than non-NIFTP lesions (mean 22.97 mm +/− 12.3 (range 10–64 mm) vs 25.88 mm +/− 11.6 (range 6–64 mm), *p* = 0.0448). In terms of nodule composition, NIFTP nodules were significantly more often solid than either complex or cystic when compared to the non-NIFTP group (93.2% vs 74.8% respectively, *p* = 0.0067). There was no significant difference in other ultrasound features between the NIFTP and non-NIFTP groups, including echogenicity, presence of echogenic foci, margins, nodule shape, presence of abnormal lymph nodes or ACR TI-RADS Category. However, none of the patients in the NIFTP group was classified as TI-RADS 5.

**Table 3:** Ultrasonographic characteristics of the NIFTP and non-NIFTP groups

Characteristics	NIFTP	Non-NIFTP	Total	*P* value
	n = 44	n = 159	n = 203	
Tumour size (mm), Mean (SD)	22.97 +/− 12.3	25.88 +/− 11.2	25.43 +/− 11.6	0.0448^a *^
*Echogenicity*				0.1585^c^
Hypoechoic	24 (54.5)	99 (62.3)	123 (60.6)	
Isoechoic	20 (45.5)	53 (33.3)	73 (36)	
Hyperechoic	0	7 (4.4)	7 (3.4)	
Solid Composition	41 (93.2)	119 (74.8)	160 (78.8)	0.0067^b *^
Partially cystic	3 (6.8)	40 (25.2)	43 (21.2)	
*Echogenic foci*				
Absent	38 (86.4)	128 (80.5)	166 (81.8)	0.2947^c^
Microcalcifications	1 (2.3)	15 (9.4)	16 (7.8)	
Coarse calcifications	5 (11.3)	16 (10.1)	21 (10.4)	
Regular Margins	40 (90.9)	131 (82.4)	171 (84.2)	0.2421^b^
Irregular margins	4 (9.1)	28 (17.6)	32 (17.8)	
Wider-than-tall Shape	43 (97.7)	157 (98.7)	200 (98.5)	0.5215^b^
Taller-than wide	1 (2.3)	2 (1.3)	3 (1.5)	
Lymph Nodes				> 0.9999^b^
Absent	44 (100)	157 (98.7)	201 (99)	
Present	0	2 (1.3)	2 (1)	
2017 ACR TIRADS				0.1585^b^
Category				
1–3	20 (45.4)	62 (39)	82 (40.4)	
4	24 (54.6)	85 (53.5)	109 (53.6)	
5	0	12 (7.5)	12 (6)	

^a^two-tailed Mann-Whitney test

^b^two-tailed Fisher’s exact test

^c^chi-square test

^*^statistically significant (*P* < 0.05)

**Fig. 1:**
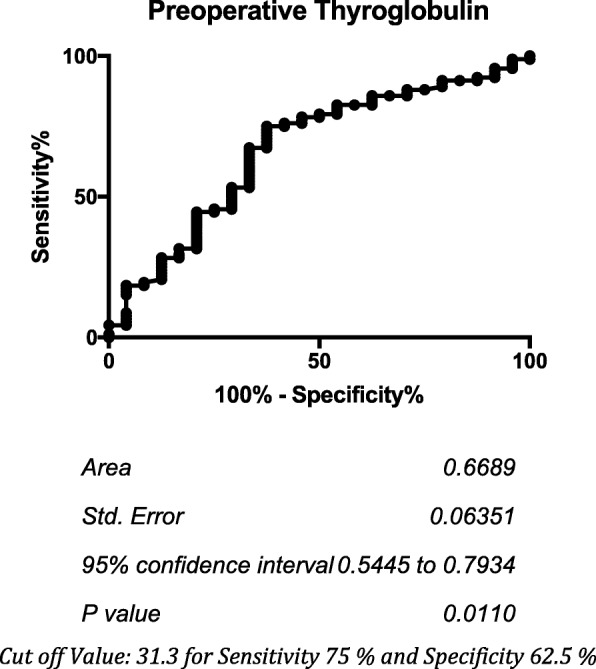
Receiver Operator Characteristics (ROC) Curve for Preoperative Thyroglobulin. *Cut off Value: 31.3 for Sensitivity 75% and Specificity 62.5%*

Measures of diagnostic accuracy were performed to determine the sensitivity, specificity, negative and positive predictive values of different Bethesda cytology categories and combination of categories to distinguish NIFTP lesions from non-NIFTP follicular cancers. No single Bethesda category or combination of categories could discriminate between both groups. Similar analysis was also performed for ACR TI-RADS category on ultrasound evaluation. As no NIFTP nodules had a category of 5 (high suspicion), while 7.5% of non-NIFTP nodules fell into this category, the combination of low and intermediate TIRADS categories [1–4] had a sensitivity and a negative predictive value of 100% to discriminate NIFTP lesions from non-NIFTP follicular cancers. No other ACR TI-RADS category or combination could discriminate between groups. Furthermore, when calculating quartiles for the distribution of preoperative thyroglobulin levels of the entire cohort and using them as categorical cut-offs, diagnostic test evaluation calculations demonstrated that values lower than the third quartile of our distribution (133.82 mcg/L) had a sensitivity of 87.50% and a negative predictive value of 89.66% to discriminate NIFTP lesions from non-NIFTP follicular cancers. Receiver-operator characteristic (ROC) curve was also plotted for preoperative thyroglobulin levels (see Fig. 1). The area under the curve was 0.67 (*p* = 0.0110). A preoperative thyroglobulin cut-off value of 31.3 mcg/L had a sensitivity of 75% and a specificity of 62.5% to distinguish NIFTP lesions from non-NIFTP follicular cancers.

## Discussion

In this retrospective study, NIFTP patients comprised 21.7% of cases initially diagnosed as FVPTC. While no single characteristic or combination of variables accurately distinguished this group from the non-NIFTP patients, some trends emerged from this analysis. Significantly more patients in the NIFTP group underwent lobectomy (50% vs.16.4%) and less received radioiodine ablation (31.8%, vs 52.2%). The NIFTP group did not differ from the non-NIFTP group in terms of mean age and gender distribution. Interestingly, the median serum Tg was significantly lower in the NIFTP group (25.55 vs 76.06 mcg/L,) and a cut-off value below 31.3 mcg/L provided the best diagnostic accuracy. This relationship was maintained after correction for the nodule size (11.34 vs 32.0 mcg/L/cm). A solid nodule composition by sonography was also more frequently found in the NIFTP group (93.2% vs 74.8%) and NIFTP nodules were significantly smaller than non-NIFTP lesions (mean 22.97 vs 25.88 mm). None of the 44 NIFTP patients was categorized as a high suspicion, ACR TI-RADS-5. Only one patient (2.3%) of the NIFTP group displayed a malignant BC-VI category and the non-NIFTP group trended to the higher risk category.

The 21.7% prevalence of NIFTP in our study is in line with the frequency of this diagnosis reported in other series (15–25%) [9–17]. Our study showed that the majority of patients were female and the mean age was around 50 years in both groups, which is consistent with most other similar studies (16, 18–19; 21). However, in the cohort reported by Singh et al. [18], the 21 NIFTP patients were significantly younger than the 153 non-NIFTP counterparts (*p* = 0.023). Our NIFTP patients had undergone lobectomy significantly more often than those with a non-NIFTP cancer, similar to the study of You et al. [19] where 51.1% of NIFTP and 30.9% of non-NIFTP had a lobectomy (*p* = 0.033). On the other hand, Kim et al. did not find any significant difference between groups with regards to surgical management or radioactive iodine therapy [20]. Since the introduction of the ATA guidelines and before the introduction of the NIFTP category, we have attempted to reduce overtreatment of low risk patients with thyroid neoplasms. This strategy may explain the more conservative management of the NIFTP patients who presented with less aggressive features.

In our study, on sonography, the internal content of nodules in the NIFTP group was significantly more often solid than complex and NIFTP nodules were smaller. These findings differ from two recent studies where there was no significant difference between groups [19, 21] for these ultrasound features. While there was no significant difference in other single ultrasound features or ACR TI-RADS score between groups in our data, You et al. [19] found that NIFTP nodules had significantly less calcifications than non-NIFTP nodules. Hanh et al. [16] found that NIFTP lesions were more often iso- or hyperechogenic, had regular margins more often, had lower rates of calcifications and had a lower K-TIRADS score. These diverging findings emphasize the need for large-scale studies to clarify the sonographic features of NIFTP vs FVPTC.

Hirokawa and colleagues [21] found that NIFTP nodules had higher rates of low suspicion ATA Category on ultrasound and lower rates of high suspicion ATA category than non-NIFTP. Measures of diagnostic accuracy in our study showed that ACR TI-RADS nodule category of 1–4 on ultrasound features had a negative predictive value and a sensitivity of 100% for NIFTP, concordant with results in the series of You et al. [19].

Altogether, we did not find a significant difference between NIFTP and non-NIFTP nodules with regards to Bethesda diagnostic category on the FNA, similarly to another series [21]. However, three recent studies found a significantly higher proportion of Bethesda IV cytology in NIFTP nodules than non-NIFTP [18–20] and one series found a significantly lower rate of Bethesda VI (malignant) cytology in NIFTP patients [18]. In our study only one patient in the NIFTP group (2.3%) displayed a malignant BC-VI category and the non-NIFTP group trended to the higher Bethesda categories.

In our data, 31.8% of NIFTP patients had a preoperative FNA showing Bethesda V (suspicious for malignancy), which is in line with most studies where the rate varied from 22.2–32% (16; 18–19). However, it was lower in two other studies (14% in Singh et al. [18] and 9.8% in Hirokawa et al. [21]) Nonetheless, in all those studies, as in our data, there was no significant difference between the rate of Bethesda V (suspicious for malignancy) cytology in NIFTP vs non-NIFTP lesions. Although Bethesda V cytology might lead to a total thyroidectomy in certain institutions, this finding did not impact the higher rate of lobectomy in our NIFTP patients (including those with Bethesda V) compared to non-NIFTP patients, similarly to the cohort by You et al. [19]

To our knowledge, no other series reported on the role of serum Tg in the distinction of NIFTP compared to non-NIFTP. Our study showed a significantly lower preoperative thyroglobulin level, without anti-Tg antibody interference, in the NIFTP group. This relationship was preserved after accounting for the TSH levels and correction for the size of the primary tumour. This finding would suggest a relationship of Tg with an increased tumour burden in the non-NIFTP group. This finding is in line with a previous retrospective review by Sands et al. [22] in which 68 patients who underwent thyroidectomy for a nodule with an indeterminate cytology (Bethesda III-V), mean preoperative thyroglobulin levels were significantly higher in patients with well-differentiated thyroid cancer (223 mcg/L) compared to those with a benign pathology (53 mcg/L), suggesting that preoperative thyroglobulin is a useful aid when making management decisions in patients with indeterminate cytology. Lee et al. also found, in a study of 164 patients who underwent thyroid surgery for a nodule with indeterminate cytology that elevated preoperative thyroglobulin increased cancer probability significantly in multivariate analysis. A preoperative thyroglobulin of 187.5 ng/mL had a specificity of 90.1% for malignancy [23]. Similarly, Trimboli et al. [24] conducted a systematic review on the role of preoperative measurement of serum thyroglobulin to predict malignancy of thyroid nodules and they found that in most studies, there was a statistically significant difference in mean or median serum thyroglobulin between patients with well-differentiated cancer and benign pathology.

Inherent to retrospective studies, ours has a number of limitations. First, given the recent description of the entity ‘NIFTP’ in 2016, even with the revision of pathology specimens from prior years, overall we had a small sample size, which hinders the power and generalizability of our findings. Moreover, we only included patients whose specimens were interpreted at our institution, and a single board-certified pathologist reanalyzed samples from previous years for the presence of NIFTP. Consequently, we were unable to determine the generalizability of our findings to other non-tertiary and non-subspecialty based pathology practices. A single endocrinologist with advanced training in thyroid ultrasound performed the initial and revised sonographic evaluation, thus there may have been intra-observer variability and possible ascertainment bias. We only included NIFTP cases with nodules where the FNA biopsy yielded Bethesda III or higher cytology. Therefore, further studies including thyroidectomy cases where the nodules had Bethesda I or II cytology may increase the number of NIFTP cases and study power. Follicular adenomas were excluded from the analysis in this protocol, but studies comparing preoperative features of three distinct groups, namely follicular adenomas as opposed to NIFTP and FVPTC would be an interesting avenue for further research. Finally we did not consider the degree of nuclear atypia in the separation of NIFTP from non-NIFTP cases as it has been reported in a meta- analysis [25]. Overall, prospective larger scale studies are needed to confirm our findings.

## Conclusion

With the growing trend towards individualized care of thyroid cancer and the goal of reducing overtreatment of low risk neoplasms, we have reported here the preoperative features that may help identify NIFTP lesions and spare the patient a total thyroidectomy. Lower preoperative thyroglobulin levels, smaller nodule size and solid sonographic texture as well as ACR TI-RADS Category of 1–4 and Bethesda cytology category III, IV and V, are more strongly associated with NIFTP than non-NIFTP follicular cancers and may favour less invasive surgical options such as lobectomy. Our data did not reveal any significant difference in other ultrasound features between the NIFTP and non-NIFTP groups, including echogenicity, presence of echogenic foci, margins, nodule shape, presence of abnormal lymph nodes or ACR TI-RADS Category.

## Data Availability

The datasets used and/or analysed during the current study are available from the corresponding author on reasonable request.
